# WNT16 from decidual stromal cells orchestrates M2 macrophage polarization via β-catenin signaling and chromatin remodeling at the maternal-fetal interface

**DOI:** 10.3389/fimmu.2025.1712898

**Published:** 2025-12-08

**Authors:** Lingyu Chang, Jiani Guo, Xi Xue, Yang Yan, Xinyi Li, Weijie Zhao, Xiaoli Sun, Jiajia Chen, Meirong Du

**Affiliations:** 1 Laboratory of Reproduction Immunology, Obstetrics and Gynecology Hospital, Fudan University Shanghai Medical College, Shanghai, China; 2 Longgang District Maternity & Child Healthcare Hospital of Shenzhen City (Affiliated Shenzhen Women and Children’s Hospital (Longgang) of Shantou University Medical College), Shenzhen, Guangdong, China; 3 Center for Reproductive Medicine, Department of Obstetrics and Gynecology, Affiliated Hospital of Nantong University, Nantong, China; 4 Shanghai Key Laboratory of Maternal Fetal Medicine, Shanghai Institute of Maternal-Fetal Medicine and Gynecologic Oncology, Shanghai First Maternity and Infant Hospital, School of Medicine, Tongji University, Shanghai, China

**Keywords:** Wnt16, Wnt/β-catenin signaling pathway, decidual macrophages, chromatin accessibility, pregnancy maintenance, recurrent spontaneous abortion

## Abstract

**Background:**

Pregnancy maintenance requires precise immunoregulation at the maternal-fetal interface, where M2-polarized decidual macrophages (dMφ) support immune tolerance. While canonical WNT signaling is reported to regulate macrophage polarization, its specific function within the decidual immune microenvironment remains insufficiently understood. Moreover, whether individual ligands such as WNT16 can epigenetically reprogram decidual macrophage responses has yet to be elucidated.

**Methods:**

Endometrial stromal cells (ESCs) from non-pregnant women and decidual stromal cells (DSCs) from normal pregnancies (NP) and recurrent spontaneous abortion (RSA) were accessed for WNT16 expression by RT-qPCR, ELISA and immunohistochemistry. The effects of WNT16 on macrophages were examined using RNA-seq and flow cytometry in peripheral monocyte-derived macrophages (pMo) and dMφ treated with recombinant WNT16 or DSC-conditioned medium. Canonical WNT pathway was evaluated by luciferase reporter assays, western blotting (WB) and immunofluorescence. Integrated ATAC-seq and RNA-seq analyses were employed to detect the epigenomic alterations downstream of the WNT/β-catenin pathway, in which the function of DIXDC1 was further evaluated by siRNA knockdown.

**Results:**

We found that WNT16 was highly expressed in DSCs from NP women compared with ESCs and DSCs from RSA patients. WNT16 selectively promoted M2-like polarization of pMo without altering NK or T cell phenotypes. Mechanistically, WNT16 activated the WNT/β-catenin pathway in dMφ and enhanced chromatin accessibility at M2-associated loci. Integrated multi-omics analysis suggested a MYC-DIXDC1 regulatory axis downstream of WNT16. Functional DIXDC1 knockdown confirmed its role in β-catenin activation and macrophage polarization, indicating that this axis may contribute to WNT16-mediated M2 programming.

**Conclusions:**

DSC-derived WNT16 promotes M2 polarization in decidual macrophages, which involves the activation of the WNT/β-catenin pathway and the feedback of a MYC–DIXDC1 regulatory axis. Our findings reveal an essential immunoregulatory and epigenetic mechanism critical for successful pregnancy.

## 1 Introduction

Successful pregnancy necessitates precise synchronization of embryonic development with maternal immunological tolerance (1). Strikingly, 50% of conceptions fail during preclinical stages due to implantation defects, with an additional 10-20% of clinically recognized pregnancies culminating in early miscarriage (2). This pathophysiological phenomenon underscores the biological necessity of developmentally programmed maternal-fetal immune tolerance, orchestrated by specialized immune cell populations in the decidua. As the second predominant immune cell population, decidual macrophages (dMφ) coordinate critical physiological processes encompassing embryo implantation, trophoblast invasion, spiral artery remodeling, decidualization, and pathogenic microorganism clearance (3, 4). All of which reflect the functional plasticity and heterogeneity of macrophages, manifested through their M1 and M2 polarization states (5). Given dMφ differentiation during pregnancy is well-known as a dynamic and highly regulated immune process, a predominant shift toward M1-polarized macrophages contributes to early pregnancy failure (6–11). In contrast, M2 macrophages exert indispensable immunomodulatory roles in pregnancy maintenance by suppressing allogeneic fetal antigen responses through their anti-inflammatory and tissue-remodeling properties, mediated by producing immunosuppressive cytokines (IL-4 and IL-10), phagocytosing apoptotic trophoblasts (12, 13), and secreting tissue-remodeling regulators (ANGPT4 and BMP6) (14, 15). However, the upstream regulatory mechanisms underlying the differentiation process and functional plasticity of dMφ remain incompletely elucidated.

The WNT signaling pathway represents an evolutionarily conserved regulatory system, widely recognized for its pivotal roles in orchestrating embryonic development, stem cell growth and differentiation, and tissue homeostasis preservation (16). Depending on the requirement for β-catenin-mediated transcriptional activation, it is classified into the canonical WNT/β-catenin pathway and non-canonical pathways, including the WNT-planar cell polarity pathway (WNT -PCP pathway) and the WNT-calcium pathway (WNT-Ca² pathway). Multiple WNT ligands exert essential regulatory functions across gestational processes: canonical signaling mediated by WNT4 critically regulates endometrial stromal cell (ESC) decidualization and trophoblast differentiation (17, 18), while non-canonical effectors such as WNT5A enhance trophoblast viability through p42/44 MAPK-dependent mechanisms (19). Beyond maintaining physiological homeostasis through embryonic development regulation, the WNT/β-catenin pathway has emerged as a multifunctional hub integrating immuno-metabolic crosstalk (20). Mechanistically, latency-competent cancer cells exploit DKK1 secretion to epigenetically suppress WNT signaling, thereby synchronizing proliferative restraint with immune cloaking. Notably, WNT2B and WNT5A drive M2 polarization of tumor-associated macrophages, accelerating epithelial-mesenchymal transition in hepatocellular carcinoma and potentiating metastatic progression in colorectal adenocarcinoma, respectively (21, 22). While the conserved paradigm of WNT ligands in harmonizing tissue ontogeny and immune equilibrium has been partially deciphered, the precise mechanisms underlying WNT signaling-mediated regulation of dMφ phenotype and functionality remain a crucial yet underexplored domain in reproductive immunobiology.

As a bifunctional ligand of the WNT family, WNT16 governs tissue homeostasis by dynamically balancing canonical and non-canonical signaling pathways: it augments osteoblast activity while suppressing bone resorption through canonical pathway activation and simultaneously drives osteoblast differentiation via JNK-mediated mechanisms (23–25). Intriguingly, murine studies have identified spatiotemporally restricted WNT16 expression during embryo implantation (26). Combined with our previous work, it extended these observations by demonstrating that decidual stromal cell (DSC)-secreted WNT16 enhances trophoblast survival through canonical WNT/β-catenin signaling (27), thereby positioning this ligand as a proactive regulator orchestrating homeostasis during gestation. However, the mechanism of its direct regulatory capacity over phenotypic polarization in dMφ—central immune sentinels at this interface—remains unclear.

In this study, we delineated the expression profile of WNT16 in DSCs and uncovered its differential expression patterns between normal pregnancies (NP) and recurrent spontaneous abortion (RSA) patients. Functionally, we demonstrated that DSC-derived WNT16 promotes M2 polarization of dMφ through activation of the WNT/β-catenin signaling pathway, which may contribute to pregnancy maintenance. Mechanistically, integrated RNA-seq and ATAC-seq analyses demonstrated that WNT16 enhances chromatin accessibility, facilitating the transcription of M2-associated genes and WNT-related molecules. Collectively, our work elucidates a molecular paradigm wherein WNT16 induces dMφ polarization through a molecular-transcriptional-epigenetic regulatory network, providing a theoretical framework for understanding the molecular interplay underlying gestational immune adaptation and highlighting its potential relevance for developing targeted strategies against RSA and other immune-related gestational disorders.

## 2 Methods

### 2.1 Human sample collection

Decidua tissues were collected from patients with clinically normal pregnancies (n=20) and unexplained spontaneous abortions (n = 20). Normal pregnancy group comprises of women who terminated gestations voluntarily with non-medical reasons during early pregnancy (6–10 weeks, 25–35 years). While the RSA patients experienced two or more consecutive pregnancy losses (7–10 weeks, 25–40 years) with unexplained reasons. Pregnancies were confirmed through ultrasound and blood tests. Patients with miscarriage attributed to endocrine, infection, anatomical, chromosomal abnormalities were excluded.

Endometrial tissue samples (n=15) were harvested from non-pregnant women of reproductive age (<50 years old) undergoing diagnostic curettage and hysterectomy for benign diseases with preserved endometrial function. Those receiving hormone treatment in recent 6 months prior to surgery were excluded.

Umbilical cord blood samples (n=8) were collected from term pregnancies delivered by cesarean section. Samples were obtained from women (<35 years old) with uncomplicated pregnancies, no obstetric or internal/external medical comorbidities, and absence of vaginal infections. Mononuclear cells were isolated separately from umbilical cord blood (cord blood mononuclear cells, CBMCs) for downstream analyses.

This study was approved by the Human Research Ethics Committee of Obstetrics and Gynecology Hospital, Fudan University. All participants signed the written informed consent. All tissues were obtained under sterile condition and immediately transported to laboratory for cell isolation within 30 minutes in pre-cooling Dulbecco’s modified Eagle’s medium (DMEM)/F12 (Gibco, USA). Before anesthesia, maternal peripheral blood samples were collected using EDTA-treated anticoagulant tubes. Mononuclear cells were isolated from maternal peripheral blood, including peripheral blood mononuclear cells (PBMCs) and peripheral monocytes (pMo) for downstream analyses.

### 2.2 Isolation of human primary cells

Decidual tissues were washed thoroughly with phosphate-buffered saline (PBS) and minced into 1-mm^3^ pieces. Afterwards, these pieces were digested with 1.0mg/ml collagenase IV (Gibco, USA) and 300U/ml DNase I (Sigma-Aldrich, USA) at 37°C with continuous agitation for 40 min. The resulting supernatant was filtered through different mesh sizes (100, 200, and 400 mesh) and centrifuged at 1500rpm for 8 min, and the harvested cells were resuspended in DMEM/F12. The cell suspension was separated using a discontinuous percoll gradient (20%/40%/60%; GE Healthcare, USA) by centrifugation at 2500rpm for 25 min. DSCs and decidual immune cells (DICs) were collected from the 20%/40% and 40%/60% interfaces respectively. After being washed with sterile PBS, DSCs were cultured in DMEM/F12 medium supplemented with 10% FBS (Sigma-Aldrich, USA) at 37 °C in 5% CO_2_ for 24 h.

ESCs were isolated from endometrial tissues following the above-described procedures, with the exception that cells were directly resuspended in DMEM/F12 medium and plated on culture flasks after centrifugation.

PBMCs and umbilical cord blood-derived monocytes were extracted by density gradient centrifugation at 2500rpm for 30 min with Ficoll-Hypaque (Solarbio, China). From PMBCs, umbilical cord blood-derived monocytes, and DICs, pMo and dMφ were obtained through positive selection using a CD14^+^ magnetic bead sorting kit (Miltenyi Biotec, Germany).

### 2.3 RNA extraction and real-time qPCR

Total RNA was extracted using TRIzol reagent (Invitrogen, USA), and 1μg cDNA were synthesized according to the manufacturer’s instructions in a 20μL reaction system (Yeasen, shanghai, China). Real Time-qPCR (RT-qPCR) amplification analysis was conducted using SYBR Green Master Mix (Yeasen, Shanghai, China) on the ABI PRISM 7900 Sequence Detection System (Applied Biosystems, USA), with actin as an endogenous control. The primer sequences were listed in **Supplementary Table S1**.

### 2.4 Western blotting

Cell lysates were prepared using RIPA lysis buffer (Beyotime Biotechnology, China) supplemented with 1% proteinase and phosphatase inhibitors (NCM Biotech, China). After centrifugation at 12000rpm for 10 min, the supernatant was collected and boiled with 5× loading buffer (NCM Biotech, China). Proteins (20μg) were separated by 10% SDS-PAGE (Epizyme, China) and transferred onto 0.45μm PVDF membranes (Millipore, USA) for 1h. The membrane was blocked with 5% skim milk for 1h at room temperature and incubated with primary antibodies against β-catenin (1:15000, ProteinTech, USA) or WNT16 (1:4000, ProteinTech, USA) overnight at 4 °C. The following day, the membranes were incubated HRP-conjugated secondary antibody (Beyotime Biotechnology, China) in room temperature for 1 h after washing 5 times in TBS-T. Immunoreactive bands were visualized using enhanced chemiluminescence solution (Millipore) and quantified with Image J, with signal intensities normalized to GAPDH expression (1:10000, ProteinTech, USA).

### 2.5 Immunohistochemical and immunofluorescent staining

IHC detected the WNT16 protein expression in endometrial and decidual tissues from patients with NP and RSA. Briefly, the slices were deparaffinized in xylene and rehydrated in a descending gradient anhydrous ethanol. The sections underwent heat-induced antigen retrieval with 1mM EDTA, followed by natural cooling to room temperature and blocking with 3% hydrogen peroxide and 3% BSA for 10 min, respectively. Sections were incubated overnight with WNT16 antibody (1:200, ProteinTech, USA) in a humidity chamber. Subsequently, the samples were stained with secondary antibody for 1h at room temperature and then incubated with DAB substrate solution. Hematoxylin was counterstained, and neutral resin was used to seal the solution.

IF was performed to individually detect β-catenin expression in dMφ of normal and RSA pregnant women and β-catenin nuclear localization. The decidual tissue sections were incubated with a rabbit anti-human CD14 antibody (Abcam, USA) and a mouse anti-human β-catenin antibody (Abcam, USA) overnight at 4 °C. After thrice washing with Tris-buffered saline (TBS), the sections received goat anti-rabbit IgG antibody (Alexa Fluor 488) and goat anti-mouse IgG antibody (Alexa Fluor 594) then underwent DAPI nuclear staining. The acquisition of all images was performed under a microscope (Olympus BX53, Japan).

### 2.6 Enzyme-linked immunosorbent assay (ELISA)

ELISA kits (Weiao Biotech, China) were used to quantify cytokine secretion according to the manufacturer’s protocols. WNT16 levels were measured in culture supernatants of ESCs and DSCs, whereas IL-10 and IFN-γ were assessed in supernatants of monocytes treated with different concentrations of WNT16.

### 2.7 Flow cytometry (FCM)

Single-cell suspension was prepared as described previously. Cells were pretreated with activation cocktail (423303, BioLegend, USA) 6h at 37 °C in advance to detect intracellular cytokine levels. After stained with human Trustain Fc X™ (422301, BioLegend, USA) to block non-specific Fc receptor binding, the harvest activated cells were incubated with cell surface antibodies for 30min at room temperature. Added 1mL FOXP3 Fix/Perm buffer (421401, BioLegend, USA) into each sample for fixation and permeabilization at room temperature for 20min, the cells were sequentially incubated with intracellular molecules for 30min. The details of all antibodies are shown in **Supplementary Table S2**. All samples were performed using CytoFLEX (Beckman Coulter, USA), and the data were analyzed using FlowJo Version 10.8.1 software (TreeStar, USA).

### 2.8 Cytometric bead array

CD14^+^ monocytes were isolated from human umbilical cord blood using magnetic bead-based separation. Following 48-hour *in vitro* culture under the WNT16 treatment, cell-free supernatants were collected for cytokine analysis. The levels of IL-2, IL-4, IL-10, and IFN-γ were quantified using the CBA Human Th1/Th2 Cytokine Kit (Cat. No. 550749, BD Biosciences), in accordance with the manufacturer’s instructions.

### 2.9 Preparation and concentration of conditioned medium of DSC

To ensure standardized processing, DSCs were seeded at a density of 5×10^5^ per well in 6-well plates and cultured for 48h before collecting the cell supernatant. High speed centrifugation at 12000rpm for 5 min to remove cellular debris. Then the supernatants were transferred to Amicon^®^ Ultra Centrifugal Filters (Sigma-Aldrich, USA) and centrifugated at 4000rpm for 30 min to concentrate and desalt. Finally, the concentrates were reconstituted with Roswell Park Memorial Institute 1640 (RPMI-1640) (Gibco, USA) complete medium to acquire different concentrations of DSC-CM.

### 2.10 Dual-luciferase reporter assay

The 8×TOPFlash-miniP and 8×FOPFlash (TOPFlash mutant) reporter sequences were constructed and inserted into the GV238 expression vector. Following co-transfection of the reporter plasmids and pRL-TK/Renilla internal control plasmid into HEK293T cells, the cultures were stimulated with WNT16 recombinant protein (rhWNT16) (R&D Systems, USA). After 48 hours, luciferase activity was evaluated using dual-luciferase reporter analysis equipment. (Promega, USA). We divided the firefly luciferase value with Renilla luciferase value for normalizing and calculating relative luciferase activity. The sequences of the reporter plasmids have exhibited in **Supplementary Table S3**.

### 2.11 RNA interference

siRNA sequence for knockdown of CTNNB1 and DIXDC1 were commercially synthesized (Generay, China). The functional sequences were as follows:​​ CTNNB1 (Forward: AACTTGCCACACGTGCAATC; Reverse: CCCACTTGGCAGACCATCAT) and DIXDC1 (Forward: GCAGGGAUCAUUCUGGGUAAATT; Reverse: UUUACCCAGAAUGAUCCCUGCTT). A negative control sequence: (Forward: UUCUCCGAACGUGUCACGUTT; Reverse: ACGUGACACGUUCGGAGAATT) was included. Following transfection with lipo3000 reagent kit (Invitrogen, USA)-complex siRNA, cells were analyzed 48 h later. Transfected cells were used in subsequent experiments.

### 2.12 mRNA sequencing and data analysis

In this study, we processed and analyzed peripheral blood macrophages, decidual macrophages and stromal cells from decidual and endometrial tissues. Total RNA was extracted using the TRIzol regent and quantified to 1μg for further experiment. Next generation sequencing library preparations were constructed according to the manufacturer’s protocol (GENEWIZ, China). Differential expression analysis was performed via R/Bioconductor with the “DESeq2” package, applying negative binomial distribution with significance thresholds set at |Log2-fold change (log2FC) | >1 and adjusted p-value <0.05. Gene Ontology (GO) enrichment analysis utilized “GOSeq” package to identify the significantly enriched terms. Gene set enrichment analysis (GSEA) was exhibited using GSEA software (4.4.0).

### 2.13 ATAC sequencing and data analysis

ATAC-seq were conducted by Roissance Biotech (Shanghai, China). We lysed and collected nuclei following standard protocols (28). According to the manufacturer’s instructions (Vazyme, cat#TD711), high-throughput DNA sequencing libraries were constructed, incorporating transposition. Libraries underwent 150-basepair paired-end sequencing on an Illumina NovaSeq 6000 (Illmina). We sequentially employed Fastp (v0.23.4) for adapter trimming at the 3’ end and FastQC (v0.12.3) for base quality assessment.​ To obtain a genome-wide overview of open chromatin regions in each sample, peak calling was first performed using MACS2 (v2.1.2). Subsequently, Deeptools (v2.4.1) was used to calculate signal intensity in specific genomic regions, and ChIPseeker (v1.16.1) was applied to annotate the genomic features of peaks across different genomic regions. Differentially accessible regions were identified using the criteria of |log_2_FC| > 1 and P < 0.05 (WNT16 vs Control). All sequencing tracks were visualized using the Integrative Genomics Viewer (IGV, v2.19.4), and motif analysis was conducted with HOMER (v4.10).

### 2.14 Integrated analysis of ATAC-seq and RNA-seq

We combined ATAC-seq result with RNA-seq result to explore the potentially critical regulatory DEGs in the open chromatin regions and key transcription factors. We used Venn analysis to screen out the upregulated genes for further Kyoto Encyclopedia of Genes and Genomes (KEGG) enrichment analysis. The sequencing trajectories of some genes were visualized using the IGV.

### 2.15 Statistical analysis

All statistical analyses and quantification were performed using GraphPad Prism 10 (GraphPad Software, USA). Data were presented as the mean ± SEM unless otherwise stated. Significant differences among different groups were assessed using a two-tailed Student’s t-test or one-way analysis of variance (ANOVA) followed by Bonferroni’s multiple comparison tests. P < 0.05 was considered statistically significant.

## 3 Results

### 3.1 DSC-derived WNT16 expression is upregulated during normal pregnancy.

**Figure 1:**
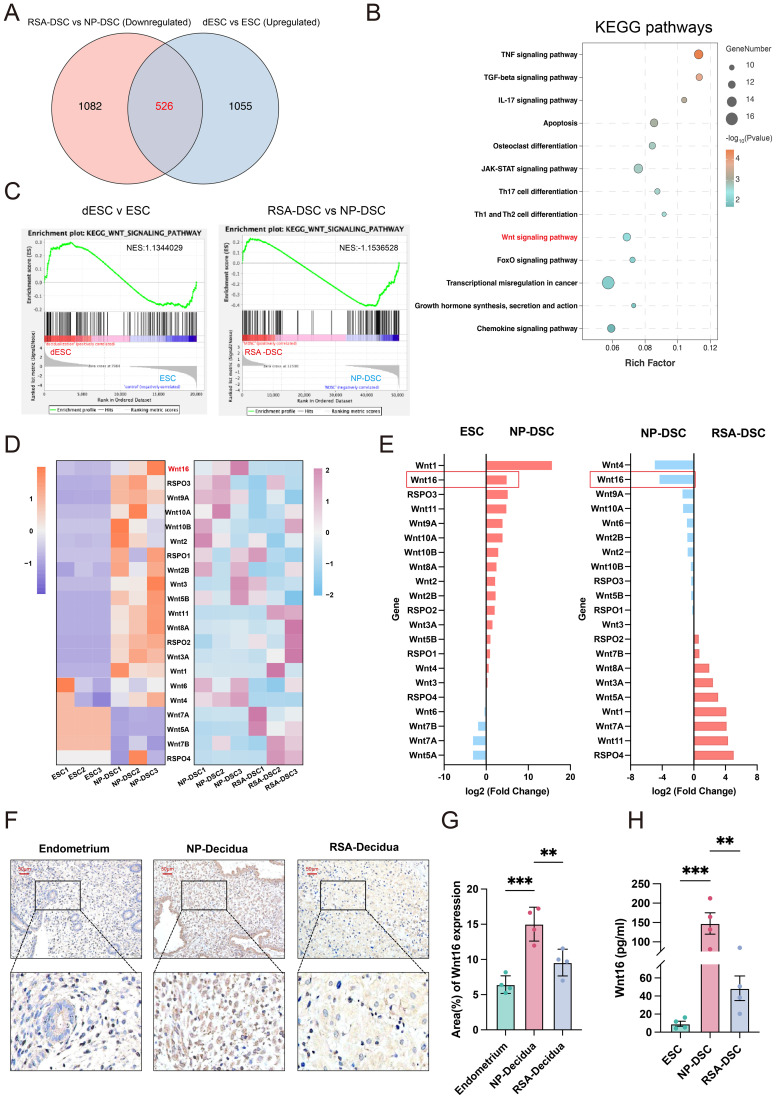
WNT16 is highly expressed in DSCs of normal early pregnancy. **(A)** Venn diagram depicting 526 genes upregulated during physiological decidualization in normal pregnancy from two datasets. **(B)** KEGG enrichment analysis was performed on the intersecting genes. **(C)** GSEA of the WNT signaling pathway based on the whole-gene ranked lists from the comparisons of dESCs vs ESCs, and RSA-DSCs vs NP-DSCs. **(D)** Heatmap showing the RT-qPCR expression patterns of WNT ligands across ESCs, NP-DSCs, and RSA-DSCs. **(E)** Bar plot displaying the log_2_fold changes (log_2_FC) in WNT ligand expression (left: NP-DSCs vs ESCs; right: RSA-DSCs vs NP-DSCs). **(F)** Representative immunohistochemical showing WNT16 expression in endometrial from non-pregnancy women and in decidual tissues from NP and RSA pregnancies (n=4), together with quantitative analysis **(G)**. **(H)** ELISA quantification of WNT16 in supernatants from 72h cultures of ESCs, NP-DSCs, and RSA-DSCs (n=4). Data are presented as mean ± SEM. **p < 0.01, ***p < 0.001.

To identify DSC-derived signaling molecules potentially involved in pregnancy maintenance, we examined two RNA-seq datasets and identified differentially expressed genes (DEGs) in two comparisons (1) ESCs from non-pregnant women versus *in vitro* decidualized ESCs (dESC), and (2) DSCs from normal pregnancies (NP-DSC) versus RSA pregnancies (RSA-DSC). We intersected 526 DEGs that were upregulated in dESC and downregulated in RSA-DSC for subsequent KEGG pathway analysis (**Figure 1A**). These overlapping DEGs were enriched in signaling pathways regulating cell differentiation and development (**Figure 1B**). Notably, the WNT signaling pathway, known for modulating embryonic development and endometrial decidualization (29), was significantly enriched. Furthermore, GSEA of all expressed genes demonstrated consistent positive enrichment of the “WNT signaling pathway” in both dESC and NP-DSC (**Figure 1C**).

To investigate which ligands might activate the WNT signaling in NP-DSC, we quantified the expression of 17 WNT family members and all R-spondins (RSPO1-RSPO4), canonical enhancers of WNT signaling (30) across the three groups (ESC, NP-DSC, RSA-DSC) using RT-qPCR (**Figure 1D**). Integrating data from two comparison groups (NP-DSC vs ESC; NP-DSC vs RSA-DSC), WNT16 exhibited a consistent alteration trend, being both the second highest among WNT ligands, with approximate 5-fold changes (**Figure 1E**). Then we examined WNT16 expression in endometrial and decidual tissues. In agreement with the transcriptional data, IHC revealed higher WNT16 expression in NP decidual tissues compared to endometrium or RSA decidual tissues (**Figures 1F, G**). ELISA quantification of WNT16 secretion levels in supernatants from ESCs, NP-DSCs, and RSA-DSCs recapitulated this expression pattern (**Figure 1H**). Collectively, these findings demonstrate the activation of the WNT signaling pathway during normal pregnancy and identify WNT16 as a highly expressed regulator in first-trimester DSCs, suggesting a potential role in pregnancy maintenance.

### 3.2 WNT16 polarizes macrophages to an M2-like phenotype

**Figure 2:**
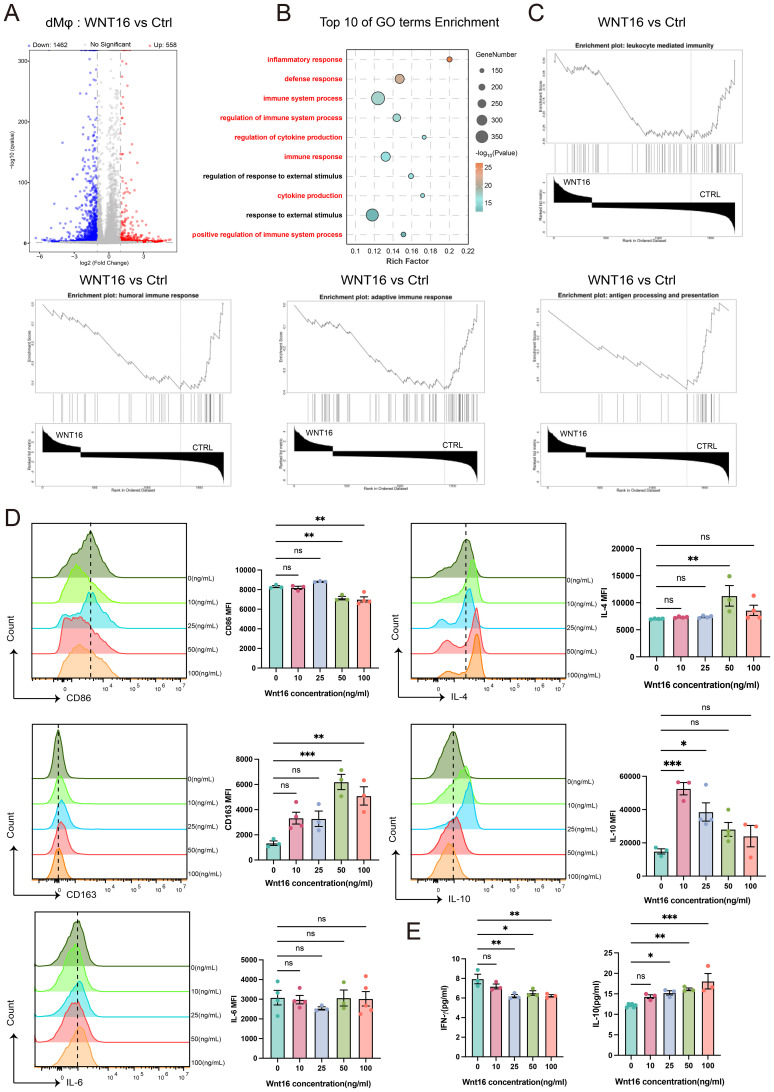
WNT16 drives toward an M2-like phenotype. **(A)** Volcano plot of DEGs in WNT16-treated pMo relative to untreated controls(∣log_2_FC∣> 1 and P< 0.05). **(B)** GO analysis of DEGs showing the top 10 enriched biological process terms, ranked by adjusted P value. **(C)** GSEA of immune-related pathways based on DEGs. **(D)** FCM analysis of representative markers in pMo after 48 hours of WNT16 treatment (n=3-5). **(E)** ELISA measurement of IL-10 and IFN-γ levels in culture supernatants from WNT16-treated pMo (n=3). Data are presented as mean ± SEM. NS, not significant; *p < 0.05, **p < 0.01, ***p < 0.001.

As a central hub at the maternal-fetal interface, DSCs play pivotal roles in orchestrating immune tolerance during early pregnancy (31). Within this context, the crosstalk between DSCs and DICs is essential for establishing maternal-fetal immune homeostasis, while its disruption is strongly associated with RSA (32, 33). Therefore, we attempted to investigate whether DSC-derived WNT16 regulates specific immune cell subsets to support a pregnancy-compatible immune environment. Firstly, recombinant human WNT16 protein was used to treat CBMCs for 48 hours, followed by FCM analysis to assess key surface markers on NK cells, T cells, and macrophages. The results showed that WNT16 had no significant effect on NK or T cells but increased the secretion of IL-4 and IL-10 from CD14^+^ monocyte-derived macrophages, indicative of a shift toward an anti-inflammatory feature (**Supplementary Figure S1-3**). Consequently, we performed RNA-seq on CD14^+^ monocyte-derived macrophages differentiated from CBMCs, treated with or without recombinant human WNT16 protein. The volcano plot (**Figure 2A**) indicated a total of 2020 DEGs, with 558 upregulated and 1462 downregulated. Both the GO and GSEA analyses, derived from the full set of DEGs, highlighted significant enrichment of immune response-related pathways (**Figures 2B, C****).** Building upon these transcriptional findings, we focused on dMφ to validate the impact of WNT16 by FCM. After 48 hours of WNT16 treatment with varying concentrations, CD14^+^ macrophages exhibited decreased expression of the proinflammatory marker CD86 and increased expression of the anti-inflammatory marker CD163. Correspondingly, intercellular IL-4 and IL-10 levels were elevated (**Figure 2D**). Furthermore, ELISA quantification showed that WNT16 induced a dose-dependent increase in IL-10 production and a concurrent decrease in IFN-γ secretion (**Figure 2E**). Together, these results show that WNT16 skews monocyte-derived macrophages toward an M2-like, anti-inflammatory phenotype, consistent with a potential role in promoting an immune-tolerant environment at the maternal-fetal interface.

### 3.3 DSC-derived WNT16 activates canonical WNT signaling pathway in dMφ

**Figure 3:**
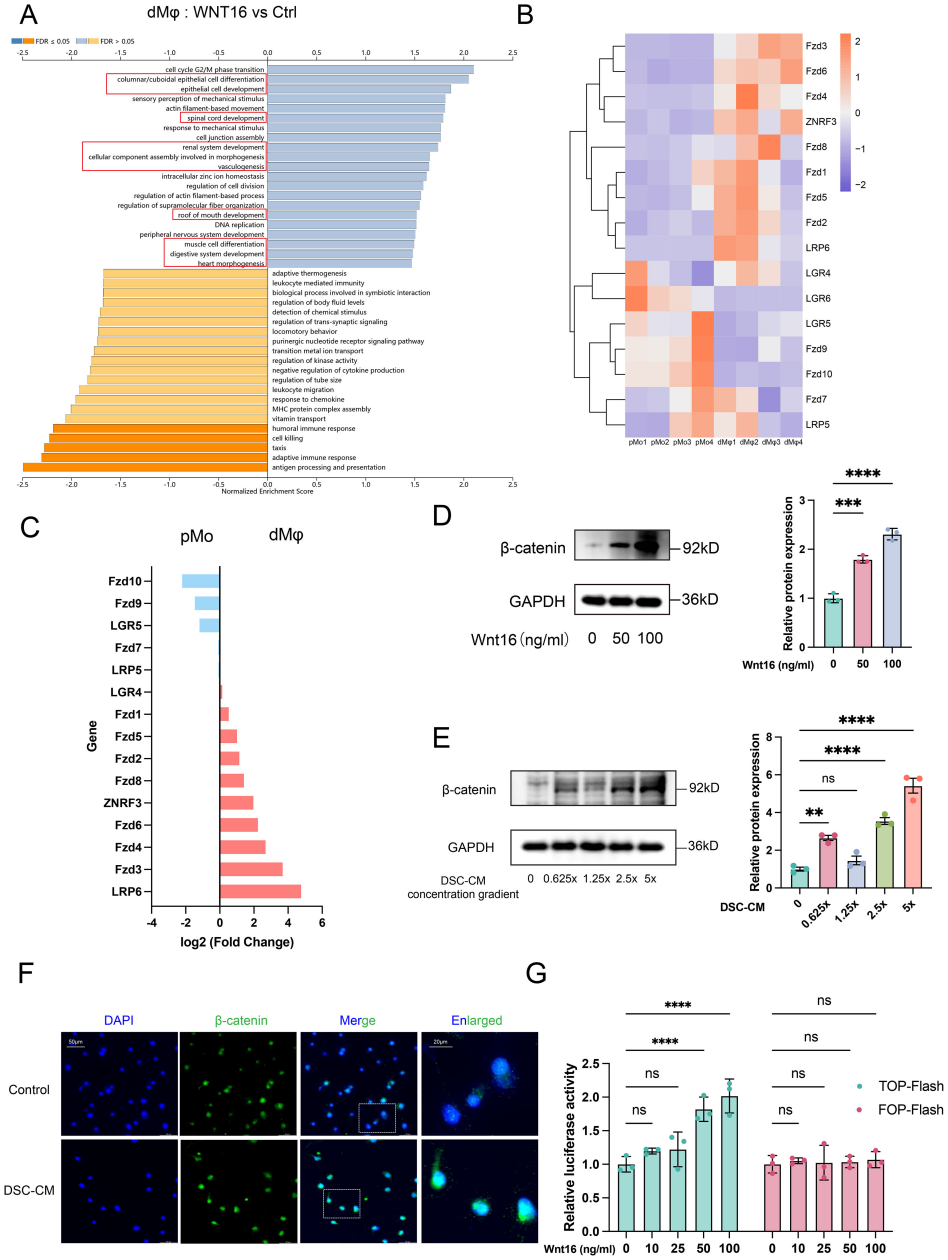
DSC-derived WNT16 activates canonical WNT signaling pathway in dMφ. **(A)** GSEA using DEGs from WNT16-treated versus control pMo, ranked by absolute NES score (|NES|). **(B-C)** Heatmap comparing mRNA expression levels of WNT receptor family members between pMo and dMφ **(B)**, with corresponding quantitative expression levels represented as log_2_FC in bar plots **(C)**. **(D-E)** Western Blot analysis of β-catenin in dMφ after WNT16 treatment **(D)** or with various concentrations of DSC-CM **(E)**, with corresponding quantification shown as bar graphs (right panels) (n=3). **(F)** Representative immunofluorescence images showing β-catenin nuclear localization in dMφ; scale bar: 20 μm. **(G)** TOP-flash and FOP-flash luciferase activity measured in dMφ after WNT16 treatment (n=3). Data are presented as mean ± SEM. NS, not significant; **p < 0.01, ***p < 0.001, ****p < 0.0001.

To explore how WNT16 affects macrophage immunomodulatory function, we performed GSEA analysis on the RNA-seq data from WNT16-treated monocyte-derived macrophages (**Figure 3A**). The analysis which performed on the ranked list of all DEGs revealed significant enrichment of pathways associated with organ development and cell differentiation, suggesting activation of the WNT signaling pathway. As a secreted protein, WNT16 requires binding to Frizzled (FZD) receptors and Low-density lipoprotein receptor-related protein (LRP) co-receptors on the cell membrane to initiate downstream intracellular signaling (34). Therefore, we next profiled the expression of multiple WNT receptors in dMφ and pMo using RT-qPCR (**Figure 3B**). Most receptors showed higher expression in dMφ compared to pMo, suggesting that dMφ may have a greater capacity to respond to WNT16 (**Figure 3C**).

To investigate the mechanism by which WNT16 exerts its effect on dMφ, we treated macrophages with recombinant human WNT16 protein and observed an upregulation of β-catenin protein expression (**Figure 3D**), a key mediator of canonical WNT signaling. Additionally, we collected the supernatant from NP-DSC, concentrated and desalted it using centrifugal ultrafiltration tubes to generate 5× DSC-CM. Treating macrophages with DSC-CM of increasing concentrations (0.625×, 1.25×, 2.5×, and 5×) resulted in enhanced β-catenin protein stability (**Figure 3E**). These data suggest that DSC-secreted WNT16 can activate the canonical WNT signaling pathway in dMφ.

We next assessed β-catenin subcellular localization using IF assay. Treatment of macrophages with 5× DSC-CM significantly increased the nuclear-to-cytoplasmic ratio of β-catenin compared to the control group (**Figure 3F**). To functionally confirm β-catenin-dependent transcriptional activation, we utilized dual-luciferase reporter assays with TOP-flash (containing TCF/LEF binding sites) and its mutant control FOP-flash plasmids. In 293T cells co-transfected with these reporters and an internal control (TK-Renilla), only high-dose recombinant WNT16 treatment significantly elevated the relative TOP-flash/FOP-flash luciferase activity (**Figure 3G**). Collectively, these findings demonstrate that DSC-derived WNT16 activates the canonical WNT signaling pathway in dMφ by promoting β-catenin stabilization, nuclear translocation, and subsequent TCF/LEF-dependent transcriptional activity.

### 3.4 β-catenin-dependent WNT signaling promotes dMφ M2 polarization and is attenuated in RSA

**Figure 4:**
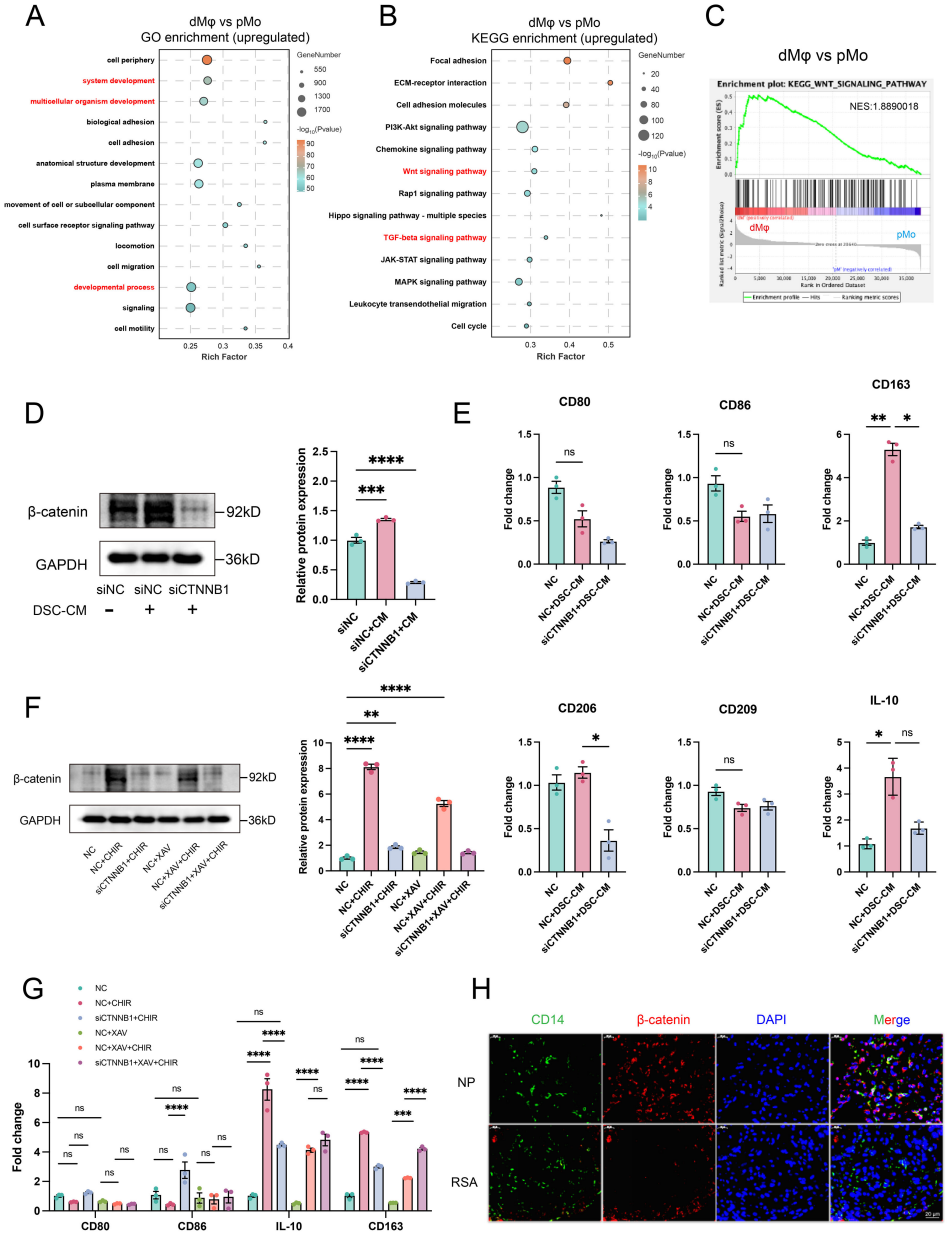
β-catenin-dependent WNT signaling mediates dMφ M2 polarization and shows reduced expression in RSA. GO **(A)** and KEGG **(B)** analyses of upregulated DEGs in dMφ versus pMφ; GSEA **(C)** performed using all DEGs to access WNT signaling pathway enrichment. **(D)** Western Blot analysis of β-catenin in dMφ following DSC-CM treatment and/or siCTNNB1 transfection (left) with quantification shown as a bar graph (right) (n=3). **(E)** RT-qPCR analysis of relative mRNA levels of macrophage polarization markers in dMφ under DSC-CM treatment and/or siCTNNB1 transfection (n=3). **(F)** Western Blot analysis of β-catenin expression in dMφ under indicated treatments(left); with corresponding quantification shown as a bar graph (right) (n=3). **(G)** The relative expression levels of macrophage polarization markers in dMφ were determined by RT-qPCR under indicated treatments (n=3). **(H)** Double immunostaining images of β-catenin (Red) and CD14 (Green) in dMφ from NP and RSA patients, with DAPI labeling nuclei (Blue). Data are presented as mean ± SEM. NS, not significant; *p < 0.05, **p < 0.01, ***p < 0.001, ****p < 0.0001.

Given that WNT16 activated the canonical WNT signaling in macrophages, we hypothesized that this pathway may participate in the phenotypic differentiation of dMφ. To test this, we performed comparative RNA-seq analysis between pMo and dMφ. GO enrichment analysis of the upregulated DEGs in dMφ revealed predominant involvement of “developmental process” and “system development” pathways. KEGG analysis further showed significant enrichment of the “WNT signaling pathway” and “TGF-β signaling pathway” (**Figures 4A, B**). These findings suggest that developmental pathways, particularly WNT signaling, may be linked to the immunoregulatory profile in dMφ. Supporting this, GSEA confirmed the enrichment of the WNT signaling pathway in dMφ compared to pMo (**Figure 4C****).**

Since CTNNB1 encodes β-catenin (35), we knocked down CTNNB1 (siCTNNB1) in dMφ to access if DSC-CM-mediated activation of WNT signaling pathway is β-catenin-dependent. To mimic the *in vivo* microenvironment, we treated dMφ with DSC-CM from NP, which contains physiologically relevant levels of endogenous WNT16. WB analysis confirmed that siCTNNB1 abolished the ability of DSC-CM to increase β-catenin protein levels (**Figure 4D**). Consistently, RT-qPCR revealed that DSC-CM treatment significantly increased the expression of M2 polarization markers CD163 and CD206, an effect that was substantially reduced by siCTNNB1 treatment (**Figure 4E**). In contrast, no significant changes were detected in the M1 markers CD80 and CD86, supporting that WNT16 primarily enhances M2-associated features rather than inducing a broad shift in macrophage polarization.

To further corroborate the role of WNT/β-catenin signaling, we employed the WNT pathway activator CHIR-99021 (CHIR) and inhibitor XAV-939 (XAV) in parallel experiments. CHIR-99021 inhibits glycogen synthase kinase-3β (GSK-3β), a negative regulator of β-catenin stability, thereby stabilizing β-catenin. Conversely, XAV-939 enhances the β-catenin destruction complex, promoting β-catenin degradation (36, 37). WB analysis demonstrated that CHIR-99021 significantly increased β-catenin protein stability in dMφ, whereas siCTNNB1 apparently attenuated this effect. In contrast, XAV-939 had a modest impact on β-catenin stability (**Figure 4F**). Supporting our hypothesis, RT-qPCR revealed elevated mRNA levels of the immunoregulatory markers IL-10 and CD163 following CHIR-99021 treatment, which were partially reversed by siCTNNB1 co-treatment. Consistent with the WB results, XAV-939 exhibited limited efficacy in modulating these cytokine and receptor expressions (**Figure 4G**). To assess the clinical relevance of this regulatory mechanism, we performed IF co-staining for CD14 and β-catenin on decidual tissue sections. Significantly reduced β-catenin expression was observed in CD14^+^ dMφ from RSA cases compared to those from NP women (**Figure 4H**), indicating attenuated WNT/β-catenin activity in dMφ during pregnancy failure. Taken together, these results indicate that β-catenin-dependent WNT signaling contributes to M2-polarized of dMφ during early pregnancy. Reduced β-catenin expression and signaling activity may correlates with pregnancy failure.

### 3.5 WNT16 remodels chromatin accessibility to promote an M2-polarized epigenetic landscape

**Figure 5:**
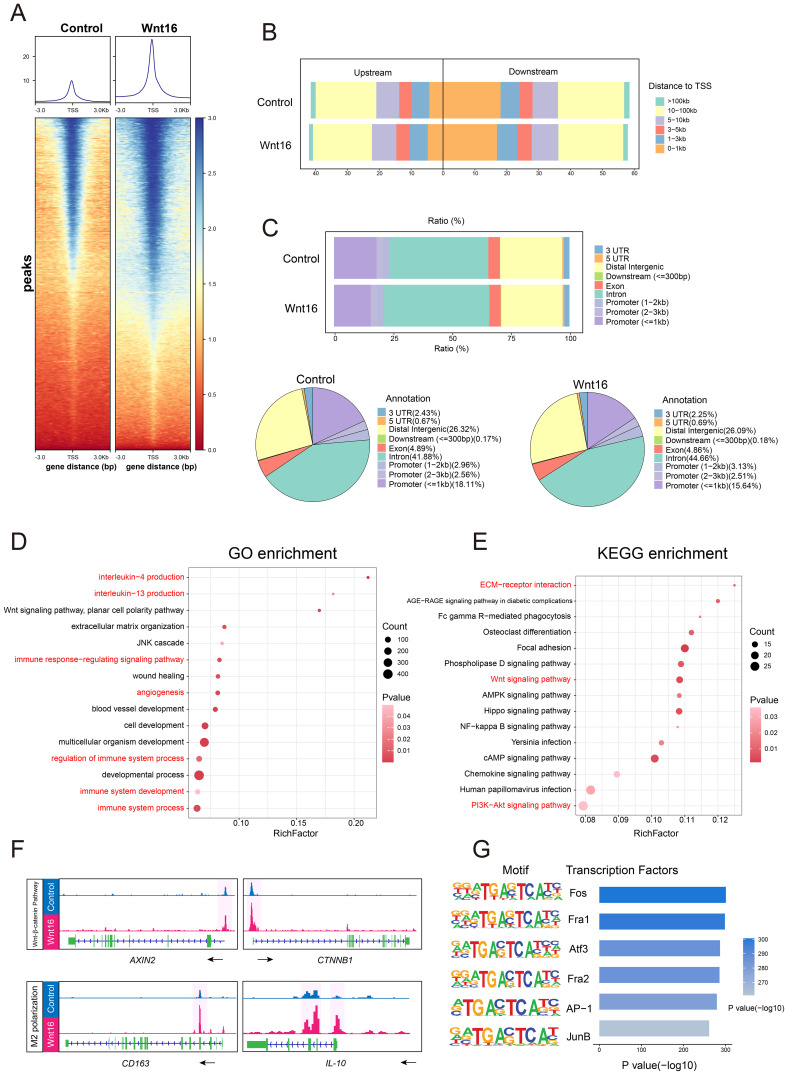
WNT16 remodels chromatin accessibility to promote an M2-polarized epigenetic landscape. **(A)** Heatmaps of chromatin accessibility in regions ±3 kb around TSS. **(B)** Genomic distribution of transcription factor binding sites in relation to TSS. **(C)** Distribution of accessible chromatin across various genomic features. GO **(D)** and KEGG **(E)** enrichment analysis of genes with upregulated chromatin accessibility in dMφ after WNT16 stimulation. **(F)** Gene tracks from ATAC-seq showing chromatin accessibility at representative WNT signaling genes (*CTNNB1*, *AXIN2*) and M2 macrophage polarization markers (*CD163*, *IL-10*) in dMφ with or without WNT16 treatment. **(G)** Top six enriched TF motifs identified in WNT16-treated dMφ by HOMER motif analysis.

Macrophage differentiation is a highly dynamic process regulated by intricate interactions among cytokines, cellular metabolism, and chromatin remodeling mechanisms (38). However, it remains unclear whether WNT16, an established immunomodulatory factor (39), mediates macrophage phenotypic reprogramming by regulating chromatin accessibility. To address this, we performed ATAC-seq on CD14^+^ monocyte-derived macrophages treated with WNT16 for 48 hours. Analysis of ATAC-seq peak enrichment within ± 3 kb of transcription start sites (TSS) revealed that WNT16 treatment induced a global increase in chromatin accessibility (**Figure 5A**). Peak distribution analysis showed that the majority of accessible regions were highly enriched within the 0–1 kb region surrounding the TSS. Notably, a significantly higher proportion of accessible chromatin regions was observed upstream of the TSS in the WNT16-treated group compared to the control group (**Figure 5B**). Annotation of peaks based on genomic distribution indicated that most accessible regions in both groups resided within intronic and intergenic regions (**Figure 5C**).

Given the global increase in chromatin accessibility induced by WNT16, we performed GO and KEGG pathway enrichment analysis specifically on genes associated with regions exhibiting increased accessibility. Notably, these genes were enriched in pathways related to M2 polarization and the WNT signaling pathway (**Figures 5D, E**). Integrative Genomics Viewer (IGV) visualization of representative M2-associated genes (*CD163* and *IL-10*) and core WNT signaling components (*AXIN2* and *CTNNB1*) showed consistently elevated ATAC-seq signal peaks in WNT16-treated groups (**Figure 5F**), suggesting that WNT16 enhances chromatin accessibility at loci relevant to M2 polarization and WNT signaling. Finally, to identify potential upstream regulators driving these chromatin accessibility changes, we performed motif enrichment analysis on the differentially accessible open chromatin regions. The top six enriched transcription factor (TF) binding motifs were primarily associated with the AP-1 family (**Figure 5G**), suggesting that AP-1–dependent regulatory elements may be engaged during WNT16-induced M2 macrophage polarization. Our findings suggest that WNT16-induced chromatin remodeling may facilitate the transcriptional activation of genes associated with M2 polarization and WNT signaling. This epigenetic reprogramming, potentially orchestrated through AP-1 family transcription factors, may contribute to the acquisition of an immunoregulatory macrophage phenotype.

### 3.6 MYC–DIXDC1 axis regulates WNT/β-catenin signaling and M2 polarization in dMφ

**Figure 6:**
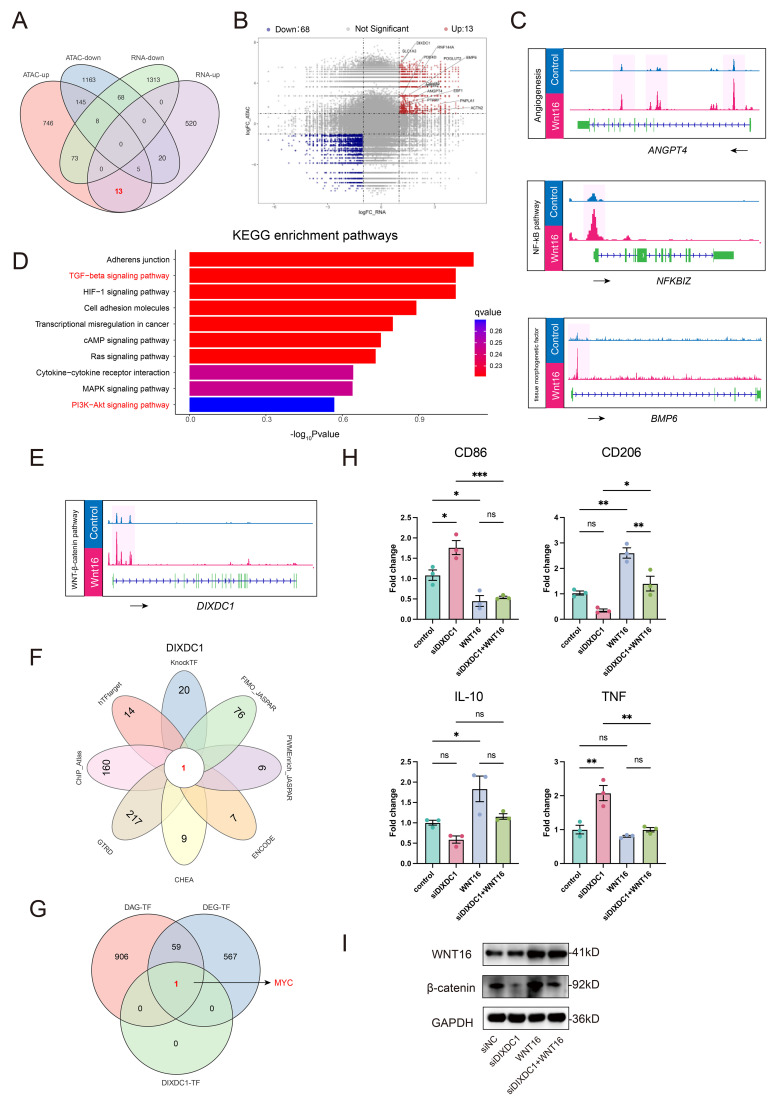
Integrated multi-omics analysis of a MYC–DIXDC1 axis in relation to Wnt/β-catenin signaling in M2 dMφ. **(A)** Venn diagram showing 13 genes commonly upregulated in both ATAC-seq and RNA-seq analyses after WNT16 treatment. **(B)** Volcano plot displays these overlapping DEGs. **(C)** IGV tracks of three M2 polarization-associated genes selected from the 13 overlapping upregulated genes. **(D)** KEGG pathway analysis of the 13 overlapping upregulated DEGs. **(E)** IGV tracks illustrating chromatin accessibility of DIXDC1. **(F)** Venn diagram showing transcription factors predicted to regulate DIXDC1 across eight databases. **(G)** Intersection of predicted transcription factors with TFs upregulated in ATAC-seq and RNA-seq analyses. **(H)** The relative expression levels of macrophage polarization markers in dMφ were determined by RT-qPCR after the treatment with siDIXDC1, WNT16 or siDIXDC1 plus WNT16 (n=3). **(I)** Western blot analysis of β-catenin and WNT16 protein expressions under the same experimental conditions. Data are presented as mean ± SEM. NS, not significant; *p < 0.05, **p < 0.01, ***p < 0.001.

In order to elucidate the downstream targets and transcriptional regulators underlying WNT16-induced chromatin remodeling, we performed an integrated analysis of the ATAC-seq and RNA-seq datasets. The Venn diagram (**Figure 6A**) identified 13 co-upregulated genes exhibiting both increased mRNA expression and enhanced chromatin accessibility following WNT16 treatment, which were individually highlighted in **Figure 6B**. Three key candidates—*ANGPT4*, *NFKBIZ*, and *BMP6*—with established roles in driving M2 macrophage polarization, showed concurrent increases in both transcript levels and chromatin accessibility upon WNT16 stimulation (**Figure 6C****) (**40–42). KEGG pathway analysis of these co-upregulated genes revealed significant enrichment in the TGF-beta signaling pathway (**Figure 6D****),** a well-known inducer of the M2-bias phenotype. These findings collectively suggest that WNT16 may orchestrate M2-like polarization through coordinated transcriptomic and epigenetic regulation of key target genes.

Of note, we observed an increase in *DIXDC1* chromatin accessibility under WNT16 stimulation (**Figure 6E**). Given that DIXDC1 is a recognized positive regulator of canonical WNT signaling (43), we hypothesized it may participate in WNT/β-catenin regulation. To investigate upstream regulators, integration of eight TF-binding databases identified MYC as a predicted transcriptional activator of DIXDC1 (**Figure 6F**), and intersection with TFs concurrently upregulated in both our ATAC-seq and RNA-seq datasets supported MYC as a candidate upstream regulator (**Figure 6G**). To functionally validate the role of DIXDC1, we performed siRNA-mediated knockdown in dMφ. siDIXDC1 treatment led to increased expression of the pro-inflammatory marker TNF-α, while co-treatment with WNT16 partially reversed these effects (**Figure 6H**). WB analysis showed that β-catenin protein levels were reduced by siDIXDC1, whereas WNT16 expression remained unaffected, suggesting that DIXDC1 is a downstream effector of WNT16. (**Figure 6I**). Together, these results clarify the regulatory hierarchy: WNT16 activates β-catenin, which subsequently induces DIXDC1 expression, and DIXDC1 in turn reinforces β-catenin signaling downstream, forming a forward regulatory module rather than a direct feedback to WNT16. Collectively, the integrated multi-omics analysis and functional validation support the existence of a MYC–DIXDC1 axis in dMφ, which contributes to WNT16-mediated regulation of β-catenin signaling and M2-associated transcriptional programs.

## 4 Discussion

**Figure 7:**
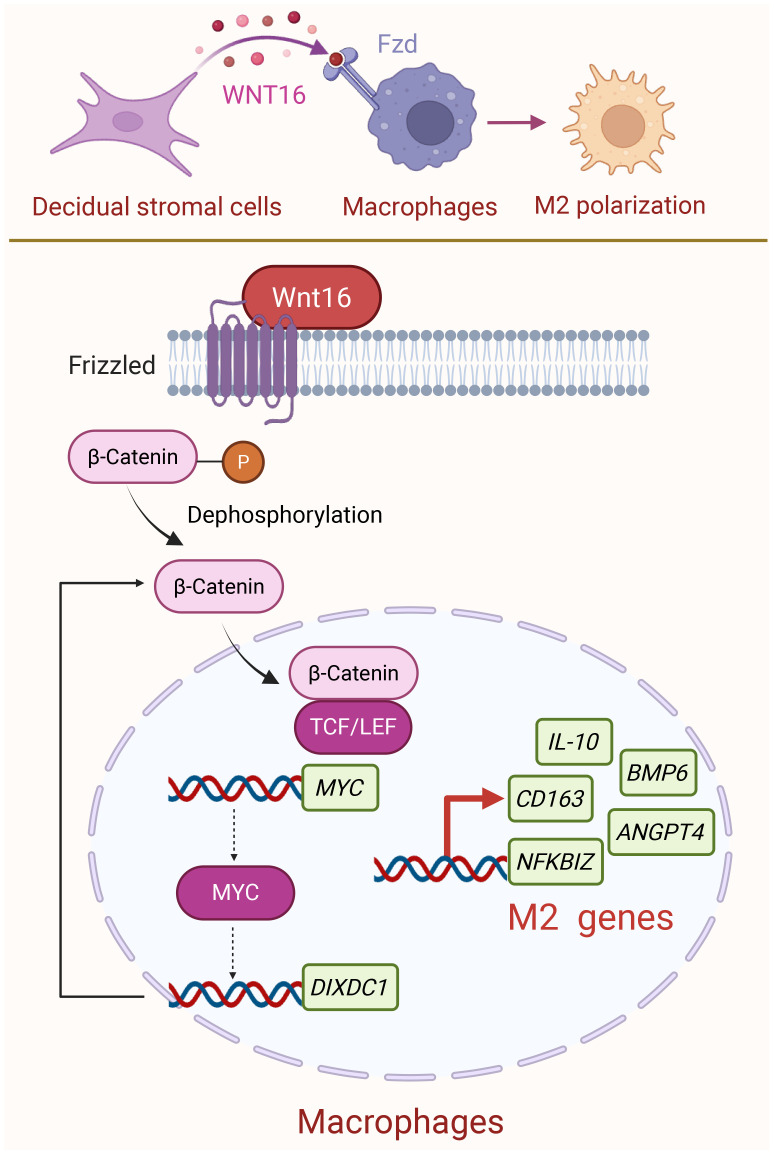
Schematic diagram of DSC-derived WNT16 promoting M2 dMφ polarization at the maternal-fetal interface. During normal pregnancy, DSCs secrete WNT16 that binds to FZD receptors on dMφ. This triggers the canonical WNT/β-catenin signaling pathway, leading to β-catenin activation and its nuclear translocation. Once in the nucleus, β-catenin may participate in transcriptional regulation together with TCF/LEF factors, potentially initiating the transcription of *MYC* and several M2 macrophage-associated genes. Subsequently, MYC could contribute to the transcription regulation of *DIXDC1*, a known activator of WNT/β-catenin pathway, which may help maintain the M2-associated transcriptional program and support an immunotolerant environment during pregnancy. Created with BioRender.com.

Multiple evidence has confirmed that the M2-polarized state of dMφ is responsible for establishing an immunosuppressive microenvironment during pregnancy (44–46). Nevertheless, despite growing interest in this area, there remain insufficient comprehensive studies exploring the molecular mechanisms. In our study, we identified a molecular mechanism whereby DSC-derived WNT16 promotes M2-like polarization of macrophages through activation of the canonical WNT/β-catenin signaling pathway. Nuclear translocation of β-catenin was associated with upregulation of *MYC* and other M2-related genes. MYC transcriptionally activates DIXDC1, establishing a MYC-DIXDC1 axis that may reinforce WNT/β-catenin signaling and support the M2-associated transcriptional program (**Figure 7**).

WNT signaling pathway exerts vital regulatory functions during pregnancy, encompassing embryo implantation and placental development. Several WNT ligands have been implicated in these processes: WNT5A promotes trophoblast proliferation and survival via the non-canonical pathway; WNT3A enhances trophoblast migration through the WNT/β-catenin signaling; and WNT2B facilitates angiogenesis by mediating trophoblast–endothelial cell interactions (19, 47, 48). However, few studies have focused on the immunoregulatory functions of the WNT signaling pathway at the maternal-fetal interface (49, 50), particularly with regard to investigating key ligands and their regulatory mechanisms. Our research found that WNT16 expression was significantly upregulated in both the NP-DSCs and dESCs, showing a concurrent trend and ranking as the second highest among all tested WNT ligands. WNT16 has been extensively studied in bone metabolism, where it regulates osteoblast differentiation, highlighting its potential to influence cell differentiation programs (51, 52). Given this established role in directing cellular fate, together with our transcriptomic findings and the critical importance of maternal-fetal immune tolerance for pregnancy maintenance, we hypothesized that WNT16 may similarly regulate the differentiation and functional programming of decidual immune cells. FCM analysis showed that WNT16 had no significant effect on the phenotype of NK cells and T cells, but it effectively prompted the polarization of CBMC-derived CD14+ macrophages towards an anti-inflammatory M2-like phenotype. Notably, the regulatory mechanism of WNT16 in osteoclast differentiation partially overlaps with that of macrophages in terms of developmental origin and signaling pathways (53, 54). This provides theoretical support for our proposal of a potential role for WNT16 in the modulation of macrophage function during pregnancy. Therefore, we sought to investigate the specific mechanisms by which the WNT signaling pathway regulates macrophage function.

It is well known that WNT16 is a secreted protein which functions through binding to specific receptors. Previous studies have reported the expression pattern of WNT pathway receptors at the maternal-fetal interface, primarily in stromal, epithelial, and trophoblast cells (55–57). In the present study, we extended this characterization to dMφ and found that most WNT receptors were highly expressed, suggesting that dMφ may be responsive to WNT ligands. We further performed WB, immunofluorescence, and dual-luciferase reporter assays to evaluate the effect of WNT16 on β-catenin protein stability, nuclear translocation, and transcriptional function. Activation of the canonical WNT pathway was comprehensively validated from three distinct perspectives: expression, function, and localization. However, while our data support WNT16 as a potential activator of the Wnt/β-catenin pathway in dMφ which likely facilitated by their elevated FZD receptor expression, we cannot exclude the involvement of other WNT ligands in this regulatory network. Besides, the expression level of β-catenin protein in dMφ from RSA patients was lower than that in women with NP, suggesting that the aberrant WNT/β-catenin signaling in dMφ is associated with RSA.

The differentiation of monocytes into macrophages is regulated by various factors at the transcriptional and epigenetic levels (58). Our ATAC-seq results demonstrated that WNT16 increases chromatin accessibility in macrophages and directly stimulates the transcription of M2-associated genes. While Yang et al. reported that WNT3A, an agonist of WNT/β-catenin signaling, promotes M2 gene expression in macrophages (59), our findings extend this concept by identifying a distinct upstream regulator. This represents a previously uncharacterized mechanism by which WNT16 modulates macrophage polarization at both epigenetic and transcriptional levels. HOMER motif analysis was conducted to identify potential regulatory transcription factors associated with changes in chromatin accessibility. The top six enriched TFs belonged to the AP-1 family, all of which have been implicated in macrophage polarization. Remarkably, a previous study found that Fra2 induced the M2 polarization by affecting the production of Th2 cytokines in asthma (60). JUNB and AP-1 have also been shown to facilitate M2-like immunosuppressive phonotypes (61, 62).This suggests that WNT16 stimulation may modulate the epigenetic landscape of macrophages through AP-1-mediated chromatin remodeling.

Through the integrated analysis of ATAC-seq and RNA-seq, we identified *DIXDC1* as a target gene upregulated under WNT16 stimulation. Further TF prediction combined with intersection analysis of transcriptomic data ultimately revealed MYC as a putative upstream regulator of *DIXDC1*. *DIXDC1* has previously been reported to positively regulate the WNT/β-catenin pathway by stabilizing the β-catenin complex and enhancing its nuclear signaling (43, 63). Intriguingly, MYC itself is a well-established downstream transcriptional target of the canonical WNT/β-catenin signaling pathway via TCF/LEF1 (64–66). Our findings suggest that MYC may transcriptionally activate *DIXDC1*, pointing to a potential MYC-DIXDC1 regulatory loop. Previous results have suggested that WNT16 stimulation may alter the epigenetic landscape of macrophages through AP-1-mediated chromatin remodeling. In addition, MYC appears to function as a gene-specific transcriptional regulator that may upregulate *DIXDC1* under a permissive chromatin environment. This dual regulatory mechanism, combining broader chromatin accessibility changes with targeted transcriptional control, underscores the epigenetic intricacy of WNT16-induced macrophage reprogramming. Altogether, the MYC–DIXDC1 axis emerges as a novel regulatory module within the WNT16-driven pathway, revealing a previously unrecognized mechanism that may contribute to immune regulation at the maternal-fetal interface.

In summary, this study identified a novel immunoregulatory pathway in which DSC-derived WNT16 promoted M2 macrophage polarization by activating the WNT/β-catenin signaling pathway. We also uncovered a MYC–DIXDC1-mediated positive feedback loop that may reinforce WNT/β-catenin signaling and support an immunosuppressive phenotype in macrophages. These findings shed new light on the epigenetic and transcriptional regulation of macrophage phenotypes at the maternal-fetal interface. However, several limitations should be acknowledged. Firstly, the specific receptor mediating WNT16 signaling in macrophages has not yet been conclusively identified. Secondly, although integrative transcriptional analysis suggests a potential MYC–DIXDC1–β-catenin regulatory circuit, the complete feedback loop has not been experimentally validated. Finally, the physiological relevance of WNT16 signaling in maintaining pregnancy outcomes remains to be confirmed in animal models. Addressing these gaps in future investigations will be essential to fully elucidate the therapeutic potential of WNT16 in pregnancy-related immune modulation.

## Data Availability

The original contributions presented in the study are included in the article/supplementary material, further inquiries can be directed to the corresponding authors.
