# Supplementary material for: WNT16 from decidual stromal cells orchestrates M2 macrophage polarization via β-catenin signaling and chromatin remodeling at the maternal-fetal interface
Source: Front Immunol. 2025 Dec 8;16:1712898. doi: 10.3389/fimmu.2025.1712898 (PMC12719274; doi:10.3389/fimmu.2025.1712898)
Supplement: Supplementary file 1 [file DataSheet1.pdf]

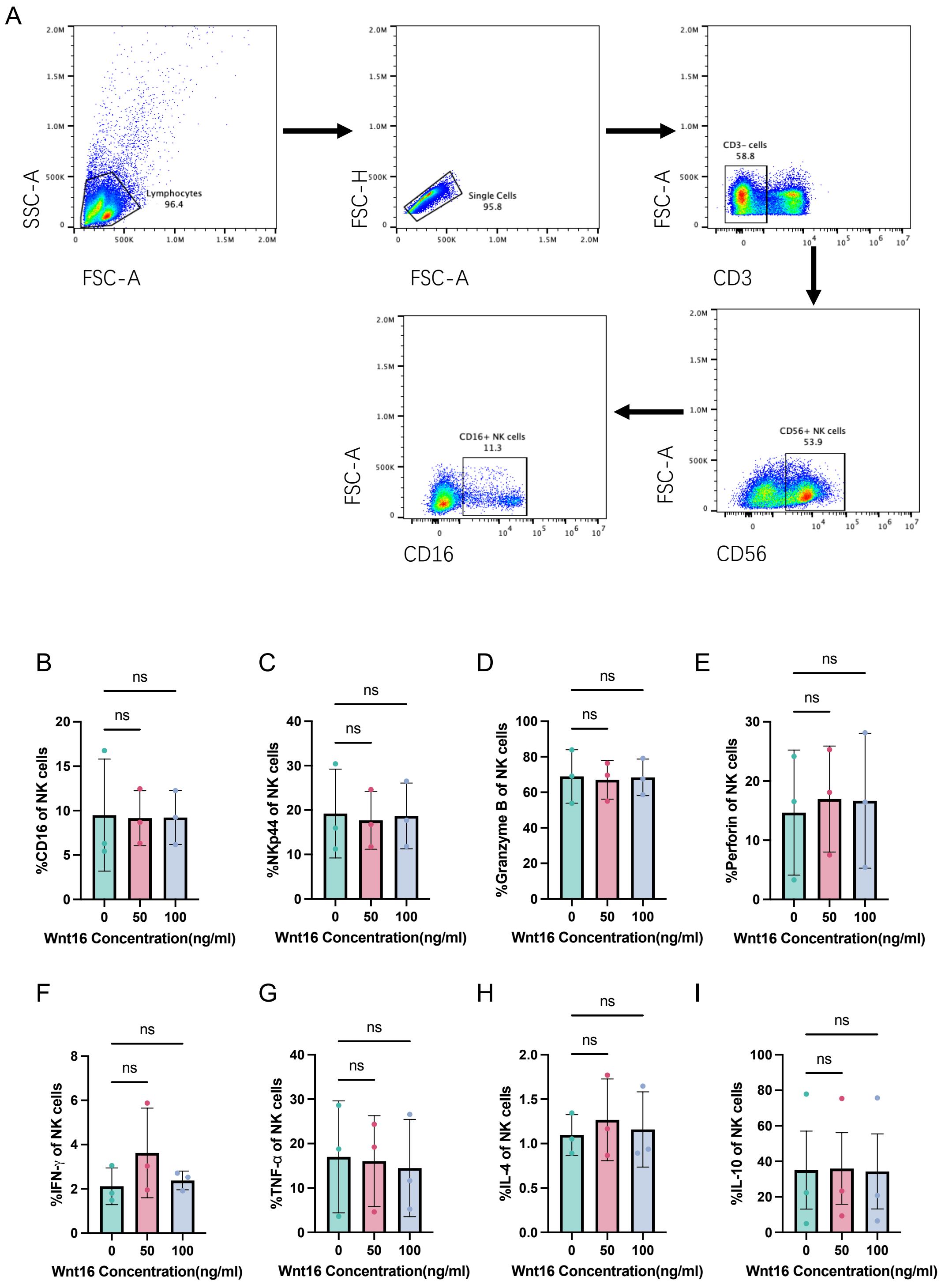

Supplementary Fig. 1

Supplementary Fig. 1 Recombinant WNT16 protein showed no significant effect on the phenotypes of NK cells. Umbilical cord blood mononuclear cells were treated with human recombinant WNT16 protein (50 ng/mL and 100 ng/mL) for 48 hours, followed by 6-hour Brefeldin A treatment prior to cell collection (n = 3). FCM was performed to gate $CD3^{-}$ $CD56^{+}$ NK cells **(A)**, and the percentages of NK cells positive for CD16 **(B)**, NKp44 **(C)**, Granzyme B **(D)**, Perforin **(E)**, IFN-γ **(F)**, TNF-α **(G)**, IL-4 **(H)**, and IL-10 **(I)** were quantified. NS, not significant.

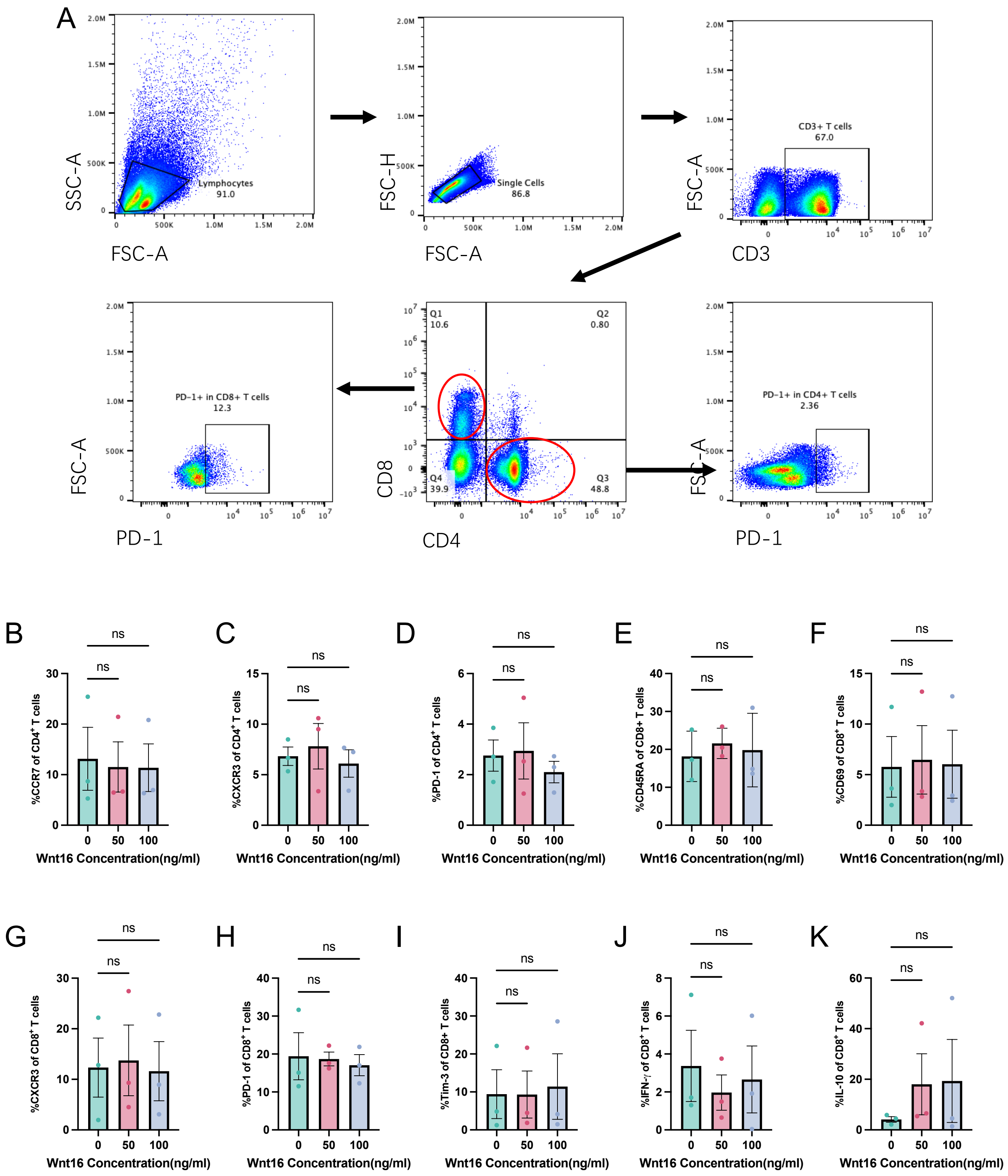

Supplementary Fig. 2

Supplementary Fig. 2 Recombinant WNT16 protein showed no significant effect on the phenotypes of CD4^{+} T cells and CD8^{+} T cells. Umbilical cord blood mononuclear cells were treated with human recombinant WNT16 protein (50 ng/mL and 100 ng/mL) for 48 hours, followed by 6-hour Brefeldin A treatment prior to cell collection (n = 3). FCM was performed to gate CD3^{+} CD4^{+} T cells **(A)**, and the percentages of CD4^{+} T cells positive for CCR7 **(B)**, CXCR3 **(C)**, and PD-1 **(D)** were quantified. Similarly, CD3^{+} CD8^{+} T cells were gated **(A)**, and the proportions of CD8^{+} T cells expressing CD45RA **(E)**, CD69 **(F)**, CXCR3 **(G)**, PD-1 **(H)**, Tim-3 **(I)**, IFN-γ **(J)**, and IL-10 **(K)** were analyzed. NS, not significant.

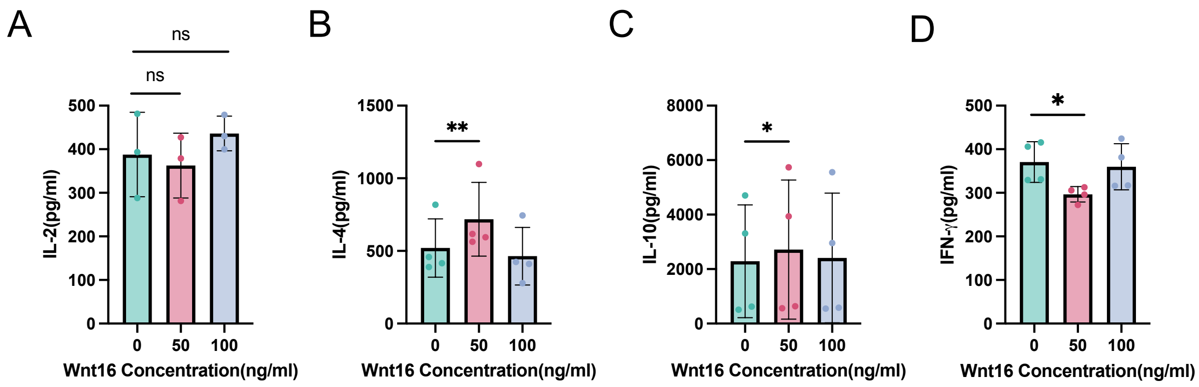

Supplementary Fig. 3

Supplementary Fig. 3 Recombinant WNT16 protein promotes the M2 polarization of CD14$^+$ monocytes. CD14$^+$ monocytes isolated from umbilical cord blood mononuclear cells were treated with human recombinant WNT16 protein (50 ng/mL and 100 ng/mL) for 48 hours (n = 3 - 4). Cytokine concentrations in the culture supernatants were quantified using Cytometric Bead Array (CBA), measuring absolute protein levels of IL-2 **(A)**, IL-4 **(B)**, IL-10 **(C)**, and IFN-γ **(D)**. NS, not significant; *$p < 0.05$, **$p < 0.01$.
