# Supplementary material for: WNT16 from decidual stromal cells orchestrates M2 macrophage polarization via β-catenin signaling and chromatin remodeling at the maternal-fetal interface
Source: Front Immunol. 2025 Dec 8;16:1712898. doi: 10.3389/fimmu.2025.1712898 (PMC12719274; doi:10.3389/fimmu.2025.1712898)
Supplement: Supplementary file 2 [file DataSheet2.docx]

Supplementary Material

# Supplementary Table

## Supplementary Table 1

**Human Primer sequences.**

| Gene | Forward sequence | Reverse sequence |
| --- | --- | --- |
| Wnt1 | ACCTCTTCGGCAAGATCGTC | TCACACGTGCAGGATTCGAT |
| Wnt2 | CACTGTGGCTAACGAGAGGT | GAAGTCAGGTTGCACACACG |
| Wnt2B | CCGAGAGTGTCAGCACCAAT | TGGACTACCCCTGCTGATGA |
| Wnt3 | CTTCGGCGTGTTAGTGTCCA | CGTGGCACTTGCATTTGAGG |
| Wnt3A | GTGGAACTGCACCACCGT | CTGCACATGAGCGTGTCACT |
| Wnt4 | CATGAGTCCCCGCTCGTG | TCCATGACTTCCAGGTTCCG |
| Wnt5A | GCAGCACTGTGGATAACACC | TCACCGCGTATGTGAAGGC |
| Wnt5B | AAGGAGTTTGTGGATGCCCG | GTGGCATTTGCAGGCTACG |
| Wnt6 | GTGCAACTGCACAACAACGAG | TCGCGAAATGGAGGCAGCTT |
| Wnt7A | AGTACAACGAGGCCGTTCAC | GCACGTGTTGCACTTGACAT |
| Wnt7B | TCGGGCAAGAGCTCCGAGTA | GGTTGTAGTAGCCCTGCTTCTC |
| Wnt8A | GAACCTGTTTATGCTCTGGGC | CAGCGTTCCCAAGCAAACTG |
| Wnt9A | GGACTTCCACAACAACCTCGTG | TGCTTCAGATGCTTGCCCAC |
| Wnt10A | AGCCTGGAGACTCGCAACAA | CCACAGGCCTTCAGTTTGCC |
| Wnt10B | GTTCTCTCGGGATTTCTTGGA | CATGACACTTGCATTTCCGCT |
| Wnt11 | AAGAATGAGAAGGTGGGCTCC | GGTACTTACAGTGGCACCGC |
| Wnt16 | AGTATGGCATGTGGTTCAGCA | AGCGGCAGTCTACTGACATC |
| RSPO1 | CTGGAGAGGAACGACATCCG | GCCTCACAGTGCTCGATCTT |
| RSPO2 | AGAGGCCGTTGCTTTGATGA | CCCATTCGCTCCAATGACCA |
| RSPO3 | GCACGCCTATCGGATGTGAG | CTCCTTGGCAGCCTTGACTA |
| RSPO4 | CAGGAGGTCAACAGGTGCAA | TGTTCTGGTGGGCCAAAGTG |
| FZD1 | GCGCTCATGAACAAGTTCGG | AGAACTCTGGAAGCAGCGAG |
| FZD2 | GCCCTCATGAACAAGTTCGGT | TCCGTCCTCGGAGTGGTTCT |
| FZD3 | TGGTGTTCCTTGGCCTGAAG | AGGAGGTGAACAATCACGCA |
| FZD4 | GAACCTCGGCTACAACGTGA | GACTCTCTGGCCAGGCAAAT |
| FZD5 | ACACCCGCTCTACAACAAGG | TATCCAGAAGGTGGCGAACG |
| FZD6 | ATTGGAGTCTTCAGCGGCTT | CAGCCCATTCTGTGCATGTC |
| FZD7 | CGCCTCTGTTCGTCTACCTC | TCATGATGGTGCGGATACGG |
| FZD8 | AGCGAAGGGACACTTGATGG | GGAGGCTTCAATGCCAGGTA |
| FZD9 | TGACTGGGCTTTGCTACGTG | AGCCGGTCAGGAGGAAACTA |
| FZD10 | AGCCATCCAGTTGCACGAG | GAGTCGGGCCACTTGAAGTT |
| LRP5 | ACCAATAACAACGACGTGGC | TCTTGCCCATCCAGTCAACG |
| LRP6 | ACATGCCTTCAGCCAACAGA | TATGGCAATGGCATGACGGA |
| LGR4 | ACTCAAAGTTCTAACGCTCCAG | AAAGCACTCAGCCCTCGAATG |
| LGR5 | CTCCCAGGTCTGGTGTGTTG | GAGGTCTAGGTAGGAGGTGAAG |
| CD80 | GGCCCGAGTACAAGAACCG | TCGTATGTGCCCTCGTCAGAT |
| CD86 | CTGCTCATCTATACACGGTTACC | GGAAACGTCGTACAGTTCTGTG |
| IL10 | TCAAGGCGCATGTGAACTCC | GATGTCAAACTCACTCATGGCT |
| CD163 | GCGGGAGAGTGGAAGTGAAAG | GTTACAAATCACAGAGACCGCT |
| CD206 | GTGATGGGACCCCTGTAACG | CTGCCCAGTACCCATCCTTG |
| CD209 | TGCTGAGGAGCAGAACTTCC | TACTGCTTGAAGCTGGGCAA |
| TNF-α | CCTCTCTCTAATCAGCCCTCTG | GAGGACCTGGGAGTAGATGAG |
| ACTB | CATGTACGTTGCTATCCAGGC | CTCCTTAATGTCACGCACGAT |

## Supplementary Table 2

**Antibodies for flow cytometry assays.**

| Antibody | Fluorescence | Manufactory | Cat.NO |
| --- | --- | --- | --- |
| Anti-human CD14 | APC/Cyanine7 | Biolegend | 367108 |
| Anti-human CD80 | FITC | Biolegend | 375406 |
| Anti-human CD86 | Phycoerythrin-cyanin7 (PE/Cy7) | Biolegend | 374210 |
| Anti-human CD163 | PE | Biolegend | 333606 |
| Anti-human CD206 | APC | Biolegend | 321110 |
| Anti-human CD209 | PerCP/Cyanine5.5 | Biolegend | 330110 |
| Anti-human CD56 | PerCP | Biolegend | 362526 |
| Anti-human CD16 | APC/cyanin7 (APC/Cy7) | Biolegend | 360710 |
| Anti-human CD336(NKp44) | APC | Biolegend | 325110 |
| Anti-human Granzyme B | PE | Biolegend | 372208 |
| Anti-human Peforin | APC | Biolegend | 308112 |
| Anti-human IFN-γ | Phycoerythrin-cyanin7 (PE/Cy7) | Biolegend | 502528 |
| Anti-human TNF-α | Brilliant Violet 510™ (BV510) | Biolegend | 502950 |
| Anti-human IL-4 | Brilliant Violet 605™ (BV605) | Biolegend | 500828 |
| Anti-human CCR7 | Brilliant Violet 650™ (BV650) | Biolegend | 353234 |
| Anti-human CD183(CXCR3) | Phycoerythrin-cyanin7 (PE/Cy7) | Biolegend | 353720 |
| Anti-human CD279(PD-1) | Brilliant Violet 510™ (BV510) | Biolegend | 329932 |
| Anti-human CD45RA | PerCP/Cyanine5.5 | Biolegend | 304122 |
| Anti-human CD69 | APC | Biolegend | 985206 |
| Anti-human CD366(Tim-3) | PE | Biolegend | 364806 |
| Anti-human IL-10 | Brilliant Violet 421™ (BV421) | Biolegend | 501422 |
| Anti-human IL-6 | Phycoerythrin-cyanin7 (PE/Cy7) | Biolegend | 501120 |

## Supplementary Table 3

**Sequence information**

| Name | Sequence |
| --- | --- |
| 8 × TOP-Flash-miniP | GGTACCGAGCTCTTACGCGAGATCAAAGGGGGTAAGATCAAAGGGGGTAAGATCAAAGGGGCGCGAGATCAAAGGGGGTAAGATCAAAGGGGGTAAGATCAAAGGGGGTAAGATCAAAGGGGCGCGCCCGCGTGCTAGCCCGGGCTCGAGATCTAGACTCTAGAGGGTATATAATGGAAGCTCGAATTCCAGCTTGGCATTCCGGTACTGTTGGTAAA |
| 8 × FOP-Flash  (TOP-Flash  mutant) | TTACGCGAGGCCAAAGGGGGTAAGGCCAAAGGGGGTAAGGCCAAAGGGGGTAAGGCCAAAGGCGCGAGGCCAAAGGGGGTAAGGCCAAAGGGGGTAAGGCCAAAGGGGGTAAGGCCAAAGGCCCGGGCTCGAGATCTAGACTCTAGAGGGTATATAATGGAAGCTCGAATTCCAGCTTGGCATTCCGGTACTGTTGGTAAA |
